# Cloning and Characterization of Spliced Variants of the Porcine G Protein Coupled Receptor 120

**DOI:** 10.1155/2015/813816

**Published:** 2015-05-17

**Authors:** Tongxing Song, Jie Peng, Jiao Ren, Hong-kui Wei, Jian Peng

**Affiliations:** ^1^Department of Animal Nutrition and Feed Science, College of Animal Science and Technology, Huazhong Agricultural University, Wuhan 430070, China; ^2^The Cooperative Innovation Center for Sustainable Pig Production, Wuhan 430070, China

## Abstract

The polyunsaturated fatty acids (PUFAs) receptor GPR120 exerts a significant impact on systemic nutrient homeostasis in human and rodents. However, the porcine GPR120 (pGPR120) has not been well characterized. In the current study, we found that *pGPR120* had 3 spliced variants. Transcript 1 encoded 362-amino acids (aa) wild type pGPR120-WT, which shared 88% homology with human short form GPR120. Transcript 1 was the mainly expressed transcript of *pGPR120*. It was expressed predominantly in ileum, jejunum, duodenum, spleen, and adipose. Transcript 3 (coding 320-aa isoform) was detected in spleen, while the transcript 2 (coding 310-aa isoform) was only slightly expressed in spleen. A selective agonist for human GPR120 (TUG-891) and PUFAs activated SRE-luc and NFAT-luc reporter in HEK293T cells transfected with construct for pGPR120-WT but not pGPR120-V2. However, 320-aa isoform was not a dominant negative isoform. The extracellular signal-regulated kinase 1/2 (ERK1/2) phosphorylation levels in cells transfected with construct for pGPR120-WT were well activated by PUFAs, especially n-3 PUFA. These results showed that although pGPR120 had 3 transcripts, transcript 1 which encoded pGPR120-WT was the mainly expressed transcript. TUG-891 and PUFAs, especially n-3 PUFA, well activated pGPR120-WT. The current study contributed to dissecting the molecular regulation mechanisms of n-3 PUFA in pigs.

## 1. Introduction

Free fatty acids (FFAs) are well known in serving as important nutrients to provide energy for the body and play important roles in various physiological regulation as signaling molecules [1–3]. Recently, a group of G-protein coupled receptors (GPCRs) have been identified as FFA receptors which mediate FFA-induced signaling in various tissues [2–9]. In particular, GPR120, which is described as an orphan receptor originally, functions as a polyunsaturated fatty acids (PUFAs) receptor and plays critical roles in systemic nutrient homeostasis regulation [8, 10].

In human, mouse, and rat, GPR120 is expressed abundantly in intestinal tract where GPR120 mediates FFA-induced the secretion of the gut hormones (glucagon-like peptide-1 and cholecystokinin), thereby regulating appetite [5, 11]. Additionally, the expression of GPR120 is also significantly abundant in adipose tissue where it participates in lipid and glucose metabolism, as well as adipogenesis regulation [12–14]. Ligands of GPR120, including n-3 PUFAs and synthetic GW9508, reduce inflammatory and increase insulin sensitivityin a GPR120-dependent manner in mice [8]. Moreover, GPR120 is found to mediate the taste of fatty acids and FFAs-induced antiapoptosis effects [15, 16].

G*α*q/11 is well known as a coupled G protein which can improve the cytoplasmic calcium released from endoplasmic reticulum and result in activation of pathways dependent on the calcium [5, 8]. In addition, several studies have showed that GPR120 activates G*α*q/11 signaling in various cell types [5, 8, 17]. The ligands of GPR120 activate the mitogen-activated protein kinase (MAPKA) pathway, leading to phosphorylation of extracellular signal-regulated kinase 1/2 (ERK1/2) [5]. Hence, both [Ca^2+^]i and ERK1/2 activation are used as indexes to reflect the function of GPR120 [5, 13, 18].

Human GPR120 has two isoforms, a long (L) form and a short (S) form [19]. However, in mouse and rat, only one isoform of GPR120 is identified [11, 20]. Most recent lines of evidence show that the hGPR120-L and hGPR120-S have different tissue distributions and pharmacological characteristics [16, 21]. Compared with human and rodents, much less is known about the porcine GPR120 (pGPR120). In database of mRNA sequence, pigs have 3 splice variants (NM_001204766, JQ437360, and JQ437361). However, there are no relative reports to characterize pGPR120. Recently, it has been reported that feeding n-3 PUFA-enriched diets could improve the intramuscular fat content and inhibit the expression of inflammation related genes, leading to increasing the mass of skeletal muscle of pigs [22, 23]. However the detailed mechanism is largely unknown. In the current study, we presented the first evidence for tissue expression of splice variants of the pGPR120 and pharmacology of different pGPR120 isoforms. These results may provide a way to help understand the nutrichemical role of n-3 PUFA in pigs.

## 2. Materials and Methods

### 2.1. Cloning of pGPR120s into pcDNA3.1

The coding sequence (CDS) of* pGPR120s* was obtained by PCR amplification using mixed porcine complimentary DNA (cDNA) extracted from the spleen tissue of 3 Landrace barrows as templates. The CDS of* pGPR120* were inserted between restriction sites EcoRI (5′) and XbaI (3′) in pcDNA3.1 (+) (Clontech, Pato Alto, CA, USA) [21]. The primer sequences and optimal PCR annealing temperatures are listed in Table 1. The identities of all constructs were confirmed by sequencing.

### 2.2. Phylogenetic and Comparative Analysis

The amino acids sequences of GPR120s from various species were retrieved from GenBank. Then the retrieved amino acids sequences and the deduced amino acid sequences from deposited porcine CDS sequences of* pGPR120* were subjected to multiple sequence alignment using ClustalW program (http://www.ebi.ac.uk/Tools/clustalw2/) using default settings. The MEGA 5.0 program was used to reconstruct the phylogenetic trees by the Neighbor-Joining (NJ) method (http://www.megasoftware.net/).

### 2.3. Cell Culture and DNA Transfection

Isolation, maintenance, and differentiation of porcine adipose derived stem cells (ADSC) and dedifferentiated fat cells (DFAT) were performed as described [8, 12, 24]. The 3D4/2 cells (ATCC) were grown in Roswell Park Memorial Institute (RPIM) 1640 (Gibco, San Diego, CA, USA) supplemented with 2 mM L-glutamine and adjusted to contain 1.5 g/L sodium bicarbonate; 4.5 g/L glucose; 10 mM HEPES; 1.0 mM sodium pyruvate supplemented with 0.1 mM nonessential amino acids (Gibco, San Diego, CA, USA), 90%; fetal bovine serum (FBS) (Gibco, San Diego, CA, USA), 10%. PK15, IPECJ2, and HEK293T cells were grown in Dulbecco's Modified Eagle's Medium (4.5 g/L glucose) (Gibco) supplemented with 2 mM L-glutamine, 10% FBS (Gibco), penicillin (100 IU/mL), and streptomycin (100 pg/mL) in a humidified atmosphere 5% CO_2_/95% air at 37°C. Cells were grown in 75-cm^2^ flasks.

The cells were transfected at 60%–70% confluence in culture plates using Lipofectamine 2000 (Invitrogen, Carlsbad, CA, USA) following Invitrogen protocols. Briefly, both Lipofectamine 2000 (1 *μ*L) and plasmid (0.5 *μ*g) were diluted using Opti-MEM (Invitrogen) to a final volume of 50 *μ*L per well and then mixed together for 15 minutes at room temperature. Then the lipo-DNA complex was added into wells. Cells were assayed 48 h after transfection.

### 2.4. RNA Isolation, Semiquantitative RT-PCR, and Quantitative PCR (qPCR)

Total RNA was extracted from adipose, skeletal muscle, ileum, jejunum, duodenum, kidney, lung, spleen, liver, and heart of 6 Landrace barrows with Trizol (Invitrogen) following the manufacturer's instructions. Total RNA from 3D4/2, PK15, IPECJ2, ADSC, and DFAT, as well as differentiated ADSC and DFAT cells, was also isolated and extracted using Trizol reagent. The 2.5 *μ*g of RNA was reverse transcribed using First-Strand cDNA Synthesis Kit (TOYOBO, Japan) and cDNAs were stored at −20°C.

Total* GPR120* expression in various tissues was determined by semiquantitative RT-PCR. The cross intron-exon boundaries primers (GPR120-407) were designed according to the common sequence of the* pGPR120* transcripts as shown in Figure 3(a). The standard PCR program for amplification consisted of an initial denaturation step at 94°C for 4 min, followed by 35 cycles of 30 sec at 94°C, 30 sec at the indicated annealing temperature, *n* min at 72°C (*n* = size in kilobases of amplified product), and a final elongation at 72°C for 5 min. The PCR products were separated by 2% agarose gel electrophoresis (Sigma, St. Louis, MO, USA). After staining, net intensity of each band was scanned using GeneSnap system (Syngene, Cambridge, UK). The expression of* pGPR120s* was quantized after normalization of samples using intensity of *β-actin*. To further check the tissue expression of total* pGPR120* expression, we also determine the expression of total* GPR120* in culture cells, which corresponded to* pGPR120* highly expressed tissues by qPCR using a Bio-Rad CFX Connect Real-Time PCR Detection System (Bio-Rad, Richmond, CA, USA). The PK15 and IPEC-J2 are cell lines isolated from the porcine kidney and small intestine, respectively. The 3D4/2 cell is the porcine macrophage, which is one of the common cell types in spleen. ADSC and DFAT cells are preadipocytes, whereas differentiated ADSC and DFAT cells represented mature adipocytes. Gene expression levels were calculated after normalization to the standard housekeeping gene *β-actin* using the ΔΔCT method. In brief, the mean of the triplicate cycle thresholds (CT) of the pGPR120 was normalized to the mean of triplicate CT of the reference *β-actin* using the calculation formula “2^CT_*β*-actin_−CT_pGPR120_^”, which indicated a relative value as a fraction of pGPR120.

The relative expression levels of the 3 spliced variants in* pGPR120* highly expressed tissues were also detected by semiquantitative RT-PCR. The primer pair (GPR120-Variants) designed for amplification of 3 spliced variants is shown in Figure 4(a). By using this primer pair, amplification will produce 3 different fragments from 3 spliced variants. The PCR products were separated by 2% agarose gel electrophoresis or 10% polyacrylamide gel electrophoresis and detected by using ethidium bromide (Sigma) to measure the levels of* pGPR120s* mRNA from various tissues and cell lines. All the primers sequences were listed in Table 1.

### 2.5. Luciferase Reporter Assays

For luciferase reporter assays, the HEK293T cells were plated into 24-well plates. Then SRE-luc or NFAT-luc vector combined with Renilla luciferase expression plasmid (pTK) was cotransfected with expression vectors for various isoform of pGPR120 or pcDNA3.1 control plasmid. Then, 24 h after transfection, the cells were changed to the medium without FBS and maintained for at least 18 h for SRE-luc or NFAT-luc analysis. Then cells were treated with the appropriate ligand for 6 h. After treatment, cells were harvested and luciferase activity in cell extracts was determined using a luciferase assay system according to standard methods in a Dual-GLO reporter assay system (Progema, Madison, USA). Luciferase values were normalized by the Renilla values. Transfection experiments were performed in duplicate and repeated at least three times [25].

### 2.6. ERK1/2 Phosphorylation Assay

HEK293T cells were plated into 24-well plates and transfected with pGPR120-WT expression vector or pcDNA3.1 using Lipofectamine 2000. After 24 h of transfection, cells were washed 2 times and maintained in the medium without FBS for at least 18 h. Then, cells were treated with 10 *μ*M docosahexaenoic acid (DHA), eicosapentaenoic acid (EPA), *α*-linolenic acid (ALA), linoleic acid (LA), palmitic acid (PLA), or 10 *μ*M TUG-891 (Sigma) for 10 min. After treatment, cells were washed with PBS and lysed in lysis buffer (Beyotime, Shanghai, China). Lysed samples were centrifuged for 10 min at 10,000 rpm at 4°C, and the supernatant was subjected to SDS-PAGE and immunoblotting. Twenty mg of proteins/lane was separated on a 10% polyacrylamide and precast SDS gel (Bio-Rad) followed by transfer on polyvinylidene difluoride membrane (Millipore, Billerica, MA, USA). The membrane was blocked for 2 h with 5% skim milk powder (Sigma) and incubated overnight with the anti-phosphorylated ERK1/2 and anti-tubulin at a 1 : 2000 dilution. After 3 washes, the secondary antibody was added at a 1 : 10000 dilution and incubated at room temperature for 1 h. After 3 washes, the membrane was exposed by using WesternBrigh Peroxide (Advansta, California, USA) in imaging system (Carestream, New York, USA). The protein amount was normalized with the amount of tubulin as internal controls.

### 2.7. Statistical Analyses

Data were presented as means ± SD and the difference between groups was determined by Student's *t*-test.

## 3. Results

### 3.1. Molecular Characterization and Evolution of Porcine GPR120

**Figure 1:**
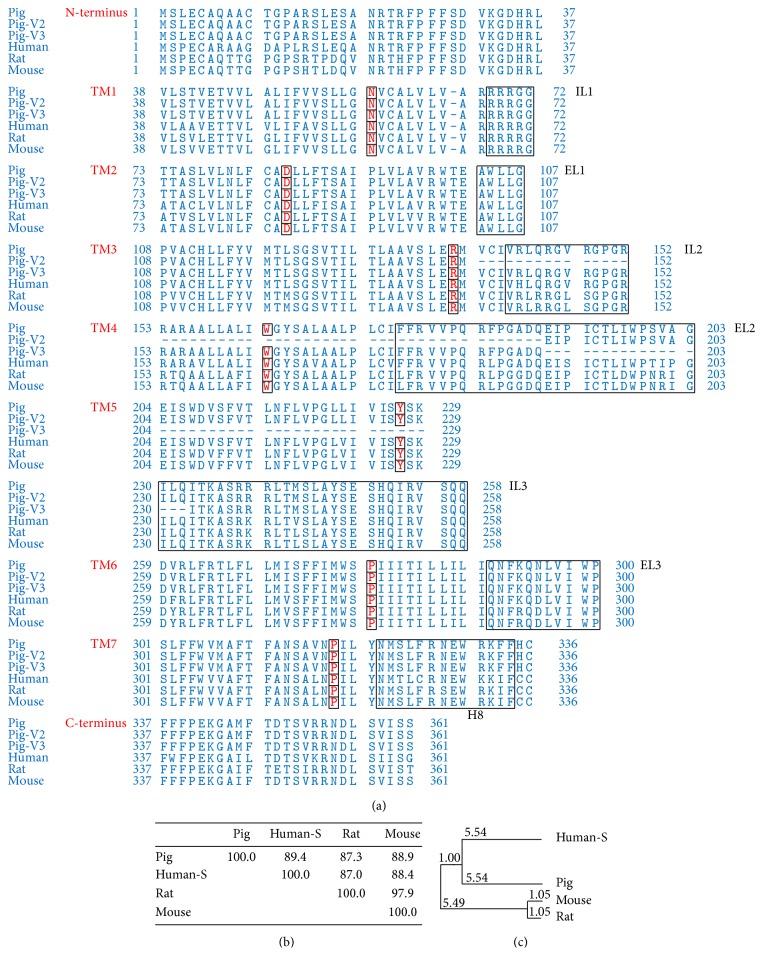
Comparison of GPR120 amino acid sequences among human, mouse, rat, and pig. (a) Alignment of the amino acid sequences among human, mouse, rat, and pig. The ELs and ILs are* boxed*. The most conserved residues in each TM are shaded in* gray*. (b) Homology and (c) phylogenetic analysis of GPR120-S amino acid sequences from human, mouse, rat, and pig. NCBI reference numbers for the proteins are human, NP_001182684.1; mouse, NP_861413.1; rat, NP_001040553.1; pig, HQ662564.1.

**Figure 2:**
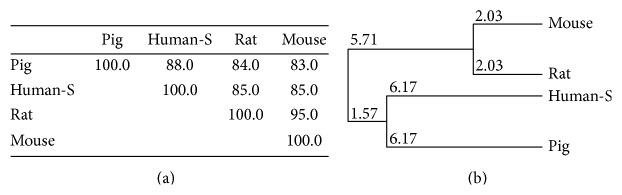
Homology (a) and phylogenetic (b) analysis of* GPR120* mRNA sequences from human, mouse, rat, and pig. NCBI reference numbers for the proteins are human, NP_001182684.1; mouse, NP_861413.1; rat, NP_001040553.1; pig, HQ662564.1.

The* pGPR120* had 3 alternative transcripts, including a 1086 bp transcript (transcript 1), a 930 transcript (transcript 2), and a 960 bp transcript (transcript 3). The transcript 1 encoded wild type pGPR120 (pGPR120-WT) of 362 amino acids (aa), whereas transcript 2 and transcript 3 encoded 310-aa and 320-aa isoforms of GPR120, respectively. The 310-aa isoform (encoded by pGPR120-V2) and the 320-aa isoform (encoded by pGPR120-V3) were the truncated isoforms, which did not contain fourth or fifth transmembrane domain, respectively (Figure 1(a)). Multiple sequence alignments and phylogenic trees comparisons of the CDS and deduced amino acid sequences of GPR120s from different species are shown in Figures 1 and 2. The pGPR120 shared higher homology (88% in CDS and 89.4% in amino acid sequence) with the human receptor sequences than they are to mouse and rat (85% and 85% in CDS; 88.4% and 87.0% in amino acid sequence), no matter for CDS or the amino acid sequence.

### 3.2. Transcriptional Expression of Total* pGPR120*

**Figure 3:**
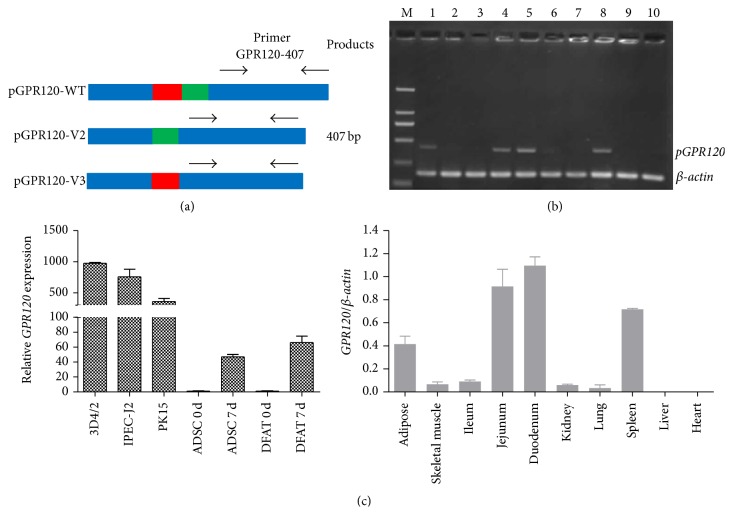
Tissue and cell expression of total pGPR120. Primer GPR120-407 was designed for the common sequence of three variants which could produce the 407 bp fragment (a). The blue domain represents the common sequence, while the red domain represents the missed sequence of V2 and the green domain represents the missed sequence of V3. PCR products of total* pGPR120* and *β-actin* were detected by 2% agarose gel electrophoresis (b). Lane 1: adipose; Lane 2: skeletal muscle; Lane 3: ileum; Lane 4: jejunum; Lane 5: duodenum; Lane 6: kidney; Lane 7: lung; Lane 8: spleen; Lane 9: liver; Lane 10: heart; M: DL2000 DNA marker. Samples were extracted from the cell lines, 3D4/2, PK15, and IPEC-J2, and the primary cells isolated from pigs, ADSC and DFAT. The expression of total* pGPR120* was determined by q-PCR (c).

The primer pair called GPR120-407 was used to detect the expression of total* pGPR120* in various tissues as Figure 3(a) shows. Semiquantitative RT-PCR analysis showed that total* pGPR120* was well detected in jejunum, duodenum, spleen, and adipose and negligibly expressed in other tissues (Figure 3(b)). To further check the expression of* pGPR120* in the corresponding established cell lines related to the highly expressed tissues, we subsequently adopted a qPCR strategy to examine the expression of total* pGPR120* in various cell lines and primary cultured cells.* GPR120* was highly expressed in adipose tissue as well as adipogenic differentiated ADSC and DFAT at day 7. In addition,* pGPR120* was also expressed in 3D4/2, IPECJ2, and PK15 (Figure 3(c)), which represent the porcine macrophage, kidney, and small intestine cells, respectively.

### 3.3. Transcriptional Expression of pGPR120 Spliced Variants

**Figure 4:**
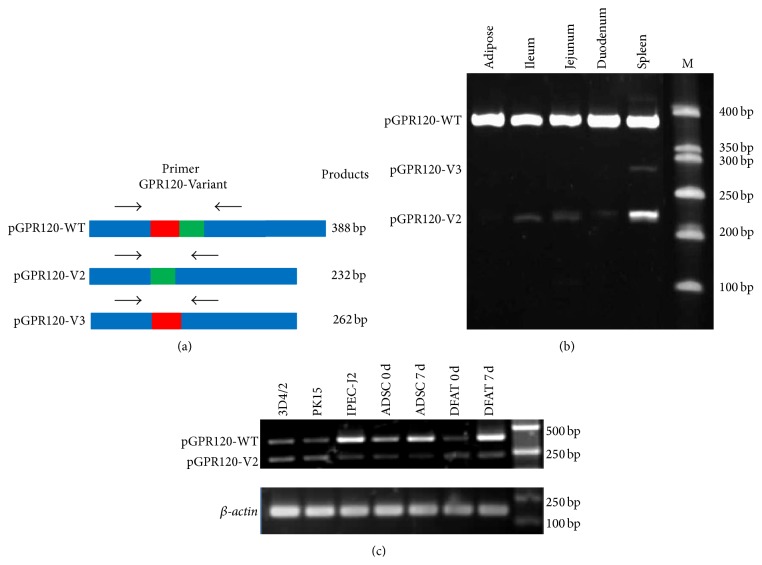
Tissue and cell expression of pGPR120 spliced variants. Primer GPR120-Variant was designed for the spliced sequence which could produce three different fragments from the variants (a). The blue domain represents the common sequence, while the red domain represents the missed sequence of V2 and the green domain represents the missed sequence of V3. The* pGPR120* spliced variants were separated by 10% polyacrylamide gel electrophoresis in adipose, ileum, jejunum, duodenum, and spleen (b). Samples were extracted from the cell lines, 3D4/2, PK15, IPEC-J2, and the primary cells isolated from pigs, ADSC and DFAT. The expression of spliced variants was detected by semiquantitative RT-PCR (c).

To investigative the tissue expression of 3 spliced transcripts in the highly expressed tissues, we designed a primer called GPR120-Variant which can detect all of the transcripts and produce different length products for 3 spliced transcripts as Figure 4(a) shows. In Figures 4(b) and 4(c), the transcript coding pGPR120-WT was expressed predominantly in ileum, jejunum, duodenum, spleen, and adipose. The pGPR120-V2 was found to be coexpressed in 3D4/2, IPECJ2, and PK15 and detected in ileum, jejunum, and duodenum, especially in spleen, but not in adipose. Another alternatively spliced transcript, pGPR120-V3, was only slightly expressed in spleen.

### 3.4. Signaling Properties of Isoforms Encoded by pGPR120 WT and V2

**Figure 5:**
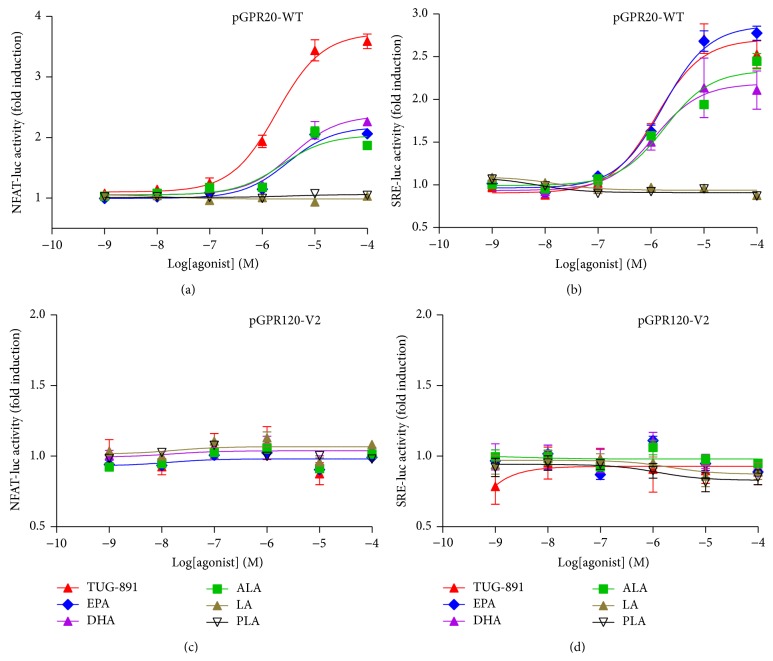
Signaling properties of pGPR120 WT and V2 isoforms. HEK293T cells were transfected with pGPR120-WT or pGPR120-V2 expression plasmid together with the NFAT ((a), (c)) and SRE ((b), (d)) reporter systems plasmids for 24 h. TUG-891 and endogenous fatty acids (EPA, DHA, ALA, LA, and PLA) were used at the range of concentration from 10^−9^ to 10^−4 ^M to treat the transfected cells with serum-free starvation for 6 h. The cells were harvested to measure the luciferase activity normalized to the Renilla values.

To identify the molecular signaling of pGPR120s, pGPR120-WT and pGPR120-V2 expression plasmid were transiently transfected into HEK293T cells with the Ca^2+^ or ERK1/2 reporter system [26]. As seen in Figure 5, under the treatment of the synthetic human and mouse GPR120 agonist TUG-891, signaling properties of pGPR120-WT and pGPR120-V2 encoded isoforms were detected as described in Section 2. The TUG-891 was used at the range of concentration from 10^−8^ to 10^−4 ^M [27]. The pGPR120-WT showed well response to the TUG-891 (EC50 = 1.88 ± 0.52 *μ*M by NFAT reporter system and EC50 = 1.25 ± 0.36 *μ*M by SRE reporter system). Besides, the WT receptor also could be well activated by endogenous free fatty acids, including EPA, DHA, and ALA but not LA or PLA. However, the pGPR120-V2 encoded isoform remained inactivated either in the NFAT or in the SRE reporter systems.

### 3.5. Isoform Encoded by pGPR120-V2 Was Not a Dominant-Negative Isoform of pGPR120

**Figure 6:**
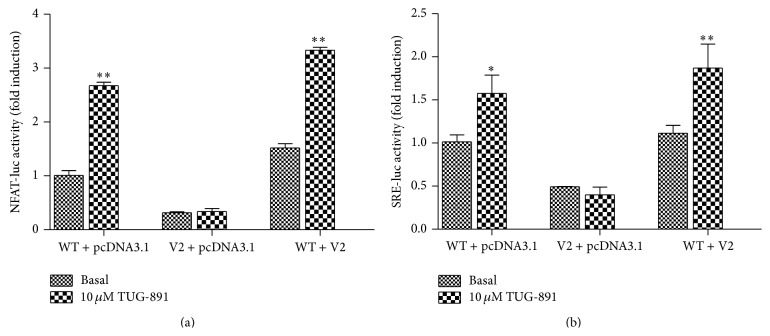
Characterization of dominant-negative pGPR120-V2. HEK293T cells were transfected as the method described. The dominant-negative pGPR120-V2 was measured by the NFAT (a) and SRE (b) reporter systems under the treatment of 10 *μ*M TUG-891. Results represent mean ± SD and ^∗^ *P* < 0.05, ^∗∗^ *P* < 0.01 compared with the control.

The alternative splicing can generate truncated GPCRs which may play a dominant-negative role in retarding the wild type receptor in the endoplasmic reticulum (ER) [28]. To explore whether pGPR120-V2 can act as a dominant-negative isoform of pGPR120-WT, we coexpressed pGPR120-V2 and pGPR120-WT at various ratios. The results showed that stimulation of TUG-891 significantly increased NFAT-Luc activity not only in cells cotransfected with pGPR120-WT and pcDNA3.1, but also in that cotransfected with pGPR120-WT and pGPR120-V2 (Figure 6). The similar result was observed by using the SRE-driven reporter system.

### 3.6. PUFAs Activates ERK1/2 Pathway via pGPR120-WT

**Figure 7:**
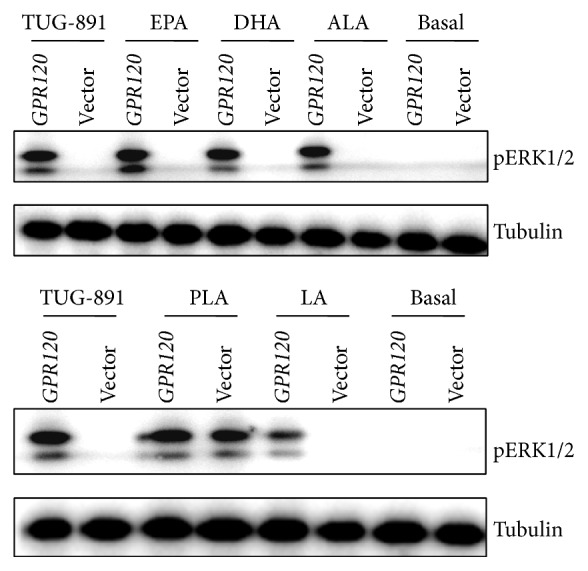
Effect of fatty acids on phosphorylation level of ERK1/2 in HEK293T cells transfected with pGPR120 expression plasmid or empty plasmid. HEK293T cells were transfected with pGPR120-WT expression plasmid or empty plasmid for 24 h. The cells were changed to the medium without FBS and maintained for at least 18 h. After a 10 min incubation of TUG-891 (10 *μ*M), ALA (10 *μ*M), EPA (10 *μ*M), DHA (10 *μ*M), LA (10 *μ*M), or PLA (10 *μ*M), cells were harvested to measure the phosphorylation level of ERK1/2.

To further evaluate the effects of pGPR120-WT on ERK1/2 signaling by nutrients, pGPR120-WT expression plasmid or pcDNA3.1 control was transiently transfected into HEK293T cells. As Figure 7 shows, TUG-891 increased the phosphorylation level of ERK1/2 compared with the basal level, as well as various PUFAs. In Figure 7, the phosphorylation level of ERK1/2 activated by TUG-891 acted as a positive group and the basal group showed no response for activation of ERK1/2. Compared with TUG-891, n-3 PUFAs had the equivalent ability to increase phosphorylation of ERK1/2, whereas n-6 PUFAs (LA) had a lower ability to increase phosphorylation of ERK1/2. It was worth noting that though PLA increased the levels of ERK1/2 activity in cell transiently transfected with pGPR120-WT expression plasmid, PLA also showed the ability to induce the phosphorylation level of ERK1/2 in the cells transiently transfected with empty plasmid, in which other PUFAs were unable to induce the phosphorylation of ERK1/2.

## 4. Discussion

As a receptor of n-3 PUFA, GPR120 is involved in a wide range of biological events, including, but not limited to, anti-inflammation and antiapoptosis in human and mouse [5, 8]. In the current study, we cloned the pGPR120 and showed that* pGPR120* had 3 transcripts. We showed that pGPR120-WT was well matched with human receptor sequence. In the present study, compared to the mouse and rat GPR120, the pGPR120-WT mRNA encodes a 362-aa protein that shared higher homology to the hGPR120. In addition, compared with human and mice, the similar expression patterns of pGPR120 in intestinal tissues maybe show the concordant functions such as inducing GLP-1 secretion [5, 12, 20] and inhibiting NF-*κ*B activity in intestinal cells [10]. The notable expressions of pGPR120 in 3D4/2 cells maybe imply the significant role of pGPR120 in macrophages such as anti-inflammation in mouse [8]. However, pGPR120 showed the low expression in lung and the abundant expression in spleen while the hGPR120 and mGPR120 were expressed highly in lung and showed low expression level in spleen [5].

Like the hGPR120, the pGPR120 also had other alternatively spliced transcripts, which were called pGPR120-V2 and pGPR120-V3 in the current study. These 3 spliced transcripts have been published in database of mRNA sequence as NM_001204766, JQ437360, and JQ437361. However, in the current study, we showed that transcript 2, which corresponds to JQ437360, only had a slight expression level in spleen. Compared with the pGPR120-V3, the notable expression of pGPR120-V2 in the spleen as well as the porcine macrophage cell 3D4/2 was observed, naturally suggesting that this alternative spliced isoform might act a role of inflammation in that tissue. Thus, the role of pGPR120-V2 in immunologic tissues and cells may be of great concern.

The distinction between the same GPCRs from various species might cause the pharmacological variation [28]. Therefore, we study the pharmacology of 2 main isoforms of pGPR120s by assaying both [Ca^2+^]i and ERK1/2 signaling. As expected, the specific agonist for human and mouse GPR120, TUG-891, can activate pGPR120-WT. However, the pGPR120-V2 almost showed negligible or no response to the small molecular agonist. It has been showed that, upon the stimulation of another human agonist GW9508, the hGPR120L also showed the poor response to the treatment while the hGPR120S can be well activated by this agonist [21]. As shown in the human GPR120 splice variants, the GPR120L inserts a splice in the key juxtamembrane between the TM domain 5 and IC3 regions, which leads to the less sensitivity of the longer receptors [21]. Compared with the pGPR120-WT, the 52 amino acids residues were deleted from the wild type amino acid sequence with the fourth transmembrane domains truncated, which may affect the normal functionality. Therefore, the truncated fragments of pGPR120, thus, suggest that the splice variant receptor may influence its receptor activity. Compared with results observed from other reports {Oh, 2010 #12}, the fold changes of the NFAT and SRE luciferase assay in the current study were lower [25]. This difference might be caused by the subtle alterations in sequence of GPR120 within different species. However, the EC50 values of treatments in the current study also range between the reliable scopes (1–10 *μ*M).

Alternative splicing acts as a main mechanism for regulating the expression of GPCR genes and allowing for the protein synthesis of truncated isoforms [28]. The truncated isoforms of some GPCRs play a pronounced role in associating with the detaining of the WT isoform in the endoplasmic reticulum (ER), which makes the attenuated expression of the WT isoform on the cell surface [28, 29]. Thus, another goal of this research was to explore whether the pGPR120-V2 is dominant-negative isoform of pGPR120. However, in our research, results showed that pGPR120-V2 had no inhibitory effects on pGPR120-WT signaling. Hence, we suggested that pGPR120-V2 was not a dominant-negative isoform. Based on the tissues and cells distributions of pGPR120-V2, the truncated isoforms showed remarkable expression in 3D4/2 and PK15 cell lines. The high expression of pGPR120 maybe implies that pGPR120 functions in these tissues and cell lines; therefore, these cell lines could be models for further research, which we did not study in depth in the present studies. Moreover, whether alternative splicing of pGPR120 in spleen contributes to regulation of pGPR120-WT expression and thereby affects the pGPR120 signaling might be interested to study.

As previously mentioned, in human and mouse, activation of GPR120 under the stimulus of the PUFAs could stimulate the ERK1/2 phosphorylation signal transduction cascade [5, 8, 10, 17]. Thus, GPR120 is named as the PUFA sensor and receptor [8]. In our studies, a series of n-3 (ALA, DHA, and EPA) and the agonist TUG-891 had similar ability in activation of ERK1/2. However, n-6 PUFA (LA) improved the phosphorylation of ERK1/2 at a lower level compared with n-3 PUFA and TUG-891, suggesting that the pGPR120-WT could also be activated by PUFAs, especially n-3 PUFA. Other reports have showed that n-6 PUFAs increase ERK1/2 phosphorylation level and even improve the mRNA expression of GPR120 [10, 30]. It is worth noting that both the n-3 and n-6 PUFAs increase ERK1/2 phosphorylation through a GPR120-dependent manner. However, PLA cannot activate either NFAT or SRE reporter gene but can increase ERK1/2 phosphorylation in cells transfected with GPR120 or control plasmid; therefore, PLA might activate ERK1/2 signaling in a GPR120-independent manner. These results match well with the results reported by Oh et al. [8]. In the study of Oh et al., they showed that the PLA increased phosphorylation level of ERK1/2 in GPR120-knockdown RAW264.7 cells and the PLA also showed no response to the SRE-luc activity in transiently transfected HEK293 cells.

Additionally, pGPR120-WT was also highly expressed in the adipose tissue and mature adipocyte, but not in the preadipocyte. This result matched well with the murine phenotype [12, 13]. Knockdown of GPR120 by siRNA could reduce the expression of adipogenic marker genes and impair the triglyceride accumulation [12]. Consequently, it implies that GPR120 may be involved in porcine adipogenesis regulation.

## 5. Conclusion

In the current study, we cloned and characterized pGPR120 for the first time. We showed that pGPR120 was expressed predominantly in ileum, jejunum, duodenum, spleen, and adipose in pigs. Although* pGPR120* has 3 spliced variants, transcript 1 which encoded pGPR120-WT was the mainly expressed transcript of* pGPR120*. In addition, pGPR120 was also the only functional isoform, which was activated by synthetic agonist (TUG-891) and PUFAs, especially n-3 PUFA. Our study contributed to understanding the molecular regulation mechanisms of PUFA in pigs.

## Figures and Tables

**Table 1:** Primers used for polymerase chain reaction.

Primer identification	Primer sequences (5′-3′)	Product size for WT (bp)	Product size for WT (bp)	Product size for WT (bp)	Annealing temperature (°C)
GPR120 full	F: GAATTCGCCACCATGGGAATGTCCCTTGAGTGC R: TCTAGACTAGCTGGAAATAACAGACAGATC	1086	930	960	62

GPR120-407	F: AAGGAGGAGGCTCACGATG R: TGACAAATAGATGCCGATAGAC	407	407	407	59

GPR120-Variant	F: GCTCTTCTACGTGATGACCCTA R: CGTGAGCCTCCTCCTTGAT	388	232	262	60

*β-actin *	F: CCAGGTCATCACCATCGG R: CCGTGTTGGCGTAGAGGT	158	—	—	60

## Abbreviations

ALA: a-Linolenic acid (C18:3n-3)
BSA: Bovine serum albumin
DHA: Docosahexaenoic acid (C22:6n-3)
EPA: Eicosapentaenoic acid (C20:5n-3)
ERK1/2: Extracellular signal-regulated kinase 1/2
GPCR: G protein coupled receptor
LA: Linoleic acid (C18:2n-6)
NFAT-RE: The nuclear factor of activated T-cell response element
PLA: Palmitic acid (C16:0)
PUFA: Polyunsaturated fatty acid
SRE: The serum response element
TM domain: Transmembrane domain.
